# PD228 Standardized Open Access Reporting Of Health Technology Assessment And Stakeholder Involvement

**DOI:** 10.1017/S0266462324004471

**Published:** 2025-01-07

**Authors:** Jack Nunn, Gillian Mason, Denny John

## Abstract

**Introduction:**

There is currently no standardized way to share information about health technology assessment (HTA). Standardised Data on Initiatives (STARDIT) can be used to overcome current limitations in sharing data about HTA processes by providing a way to report these data. This includes which stakeholders have been involved and how, the data sources used, and any impacts or outcomes observed.

**Methods:**

STARDIT development began in 2019, guided by participatory action research paradigms. A multidisciplinary international team of over 100 citizens, experts, and data users was involved in cocreating STARDIT. These cocreators included patients with cancer, people living with rare diseases, Indigenous Peoples from multiple countries, representatives involved in HTA processes, health researchers, environmental researchers, economists, librarians, and academic publishers. Methods used to involve people included public events, online discussions, and a public consultation process. STARDIT is free to use, and data can be submitted by anyone. Report authors can be verified to improve trust and transparency, and data can be checked for quality.

**Results:**

STARDIT has been used to create open access information about HTA processes that can be verified or edited by anyone at all stages of the HTA process, in multiple languages. This allows stakeholders involved in or affected by HTA processes (including patients, the public, Indigenous Peoples, and people from industry) to appraise and edit information and to self-identify the labels and terminology used to describe them. Organizations, including Australian Genomics, have recommended the use of STARDIT. Wikimedia Australia is a formal supporter and hosts data on their servers. The working beta version of STARDIT is available at ScienceForAll.World/STARDIT.

**Conclusions:**

STARDIT improves access to standardized, verified information about HTA processes, enabling well-founded comparisons of the effectiveness of different HTA methods, including the most effective methods for involving stakeholders. Since STARDIT is open access and editable by anyone, it can support participatory ways of working and help improve the equity and quality of HTA processes worldwide.

